# C-reactive protein mediates the association between leisure-time physical activity and lung function in middle-aged and older adults

**DOI:** 10.1186/s12889-019-8028-y

**Published:** 2020-01-06

**Authors:** Meng Chen, Chuanying Huang, Wenjing Feng, Yujie Li, Yili Wu

**Affiliations:** 1 Department of Epidemiology and Health Statistics, Public Health College, Qingdao University, No. 38 Deng Zhou Street, Qingdao, 266021 Shandong China; 2 Department of Geriatrics, Affiliated Hospital of Qingdao University, No. 59 Haier Road, Qingdao, 266100 Shandong China

**Keywords:** Leisure-time physical activity, C-reactive protein, Lung function, Mediating effect

## Abstract

**Background:**

Although previous studies have reported the benefits of physical activity (PA) to lung function in middle-aged and older adults, the biological mechanisms are still unclear. This study aimed to assess the extent to which C-reactive protein (CRP) mediates the association between leisure-time PA and lung function.

**Methods:**

A population-based sample was recruited from English Longitudinal Study of Ageing (ELSA), Wave 6 (2012–2013). PA was self-reported by questionnaires. CRP was analyzed from peripheral blood. Lung function parameters including forced expiratory volume in 1 s (FEV1) and forced vital capacity (FVC) were measured by using a spirometer. Baron and Kenny’s causal steps method and multiple linear regression models based on the Karlson/Holm/Bree (KHB) method were used to assess the mediating effect.

**Results:**

Among 6875 participants, 28.4% were classified into low PA, 49.8% into moderate PA, and 21.8% into high PA. Multiple linear regression models suggested that higher PA was associated with lower levels of CRP (β = − 0.048, *P* = 0.002 for moderate PA; β = − 0.108, *P* < 0.001 for high PA). CRP negatively correlated with FEV1 (β = − 0.180, *P* < 0.001) and FVC (β = − 0.181, *P* < 0.001). Higher levels of PA were associated with better FEV1 (β = 0.085, *P* < 0.001 for moderate PA; β = 0.150, *P* < 0.001 for high PA) and FVC (β = 0.131, *P* < 0.001 for moderate PA; β = 0.211, *P* < 0.001 for high PA). After introducing the CRP into the models, regression coefficients of PA with FEV1 (β = 0.077, *P* < 0.001 for moderated PA; β = 0.130, *P* < 0.001 for high PA) and FVC (β = 0.123, *P* < 0.001 for moderated PA; β = 0.188, *P* < 0.001 for high PA) decreased. The indirect effect of high PA on lung function via CRP was significant, with 9.42–12.99% of the total effect being mediated.

**Conclusions:**

The association between PA and lung function is mediated by CRP, suggesting that this association may be partially explained by an inflammation-related biological mechanism. This finding highlights the possible importance of PA in systemic inflammation and lung function, thus, middle-aged and older adults should be encouraged to enhance PA levels.

## Background

Physical inactivity is one of the primary risk factors for global mortality adding to the burden of non-communicable diseases and affecting general health [1, 2]. The World Health Organization reported that more than 80% of adolescents and 25% of adults are insufficiently physically active (PA) [1]. Lung function such as forced expiratory volume in 1 s (FEV1) or forced vital capacity (FVC) is an important predictor of morbidity of cardiovascular diseases (CVD), type 2 diabetes, cognitive disorders, disability, as well as all-cause mortality [3–7].

Epidemiological studies have demonstrated that PA was positively associated with lung function, whether in children [8–10] or adults [11–16]. The biological plausibility of the association between PA and lung function might rely on the anti-inflammatory effects of PA, which have been described in experimental studies. Regular PA produces anti-inflammatory cytokines and suppresses serum levels of C-reactive protein (CRP) and proinflammatory cytokines [17]. In this context, long-term regular PA could lead to lower basal levels of circulating inflammatory markers. Furthermore, CRP as a clinical marker of systemic inflammation can easily activate pulmonary inflammatory cells, leading to small airways damage and reduced lung function [12, 18, 19].

Although previous studies at population-level have observed the protective effect of PA on inflammation [20–25], as well as the risk effect of inflammation on lung function [26–29], few literature examined the causal link between PA, inflammation and lung function. In epidemiological studies, mediation analysis is one of the common methods to explore the biological mechanism underlying the specific exposure-disease relations. Different from moderation analysis which is used to determine whether the size or sign of the effect of exposure on disease depends on a moderator variable, mediation analysis is helpful to understand how an exposure variable affects a trait/disease through a mediator [30].

Based on a large sample derived from English Longitudinal Study of Ageing (ELSA), we hereby conducted a cross-sectional study using mediation analysis to assess the role of CRP as a mediator in the association between leisure-time PA and lung function and further to explore the extent to which CRP mediates this association.

## Methods

### Study sample

ELSA is an on-going prospective and nationally representative cohort of the English population aged 50 and over. The survey began in 2002 with 12,099 individuals. Participants were followed up every 2 years using computer-assisted personal interviews and self-completion questionnaires with additional nurse visits every 4 years for the assessment of biomarkers. Additional detail about ELSA has been previously published elsewhere [31]. ELSA was developed by a team of researchers based at the NatCen Social Research, University College London and the Institute for Fiscal Studies. The data were collected by NatCen Social Research. ELSA received ethical approval from the London Multicentre Research Ethics Committee and informed consent was given from all participants.

This research uses data from ELSA, Wave 6 (2012–2013). Participants were ineligible for lung function tests if they had eye, ear or chest surgery in the last 3 months before the assessment, or they were hospitalized with heart disease 1 month ago, or pregnant, or taking medications for the treatment of tuberculosis. Among 7014 individuals who underwent lung function tests at wave 6, 139 individuals had invalid values for their lung function measure. Finally, 6875 individuals were included in current analyses.

### Lung function

The trained nurses carried out lung function tests including FEV1 and FVC by using a spirometer (NDD Easy On-PC, ndd Medical Technologies, Inc., Massachusetts, US). Participants were asked to stand, take a deep breath and blow into the spirometer as hard and as fast as they could. Three successful measurements were taken. According to the convention for the epidemiological study, the maximum of the lung function parameters were recorded [32].

### Leisure-time physical activity

PA was self-reported in ELSA, Wave 6. Participants were asked about the frequency of mild, moderate and vigorous PA (more than once per week, once per week, one to three times a month, or hardly ever/never) during leisure time using a card to help them classify different activity intensities. Examples of mild activities included laundry and home repairs; moderate activities included gardening, moderate pace walking or cleaning the car etc. and vigorous activities included swimming or cycling, running or jogging, aerobics or gym workouts etc. We categorized PA into three groups: high (vigorous activity more than once a week); moderate (moderate activity more than once a week, or vigorous activity between once a week to one to three times a month); and the rest participants were divided into low PA [33, 34].

### C- reactive protein

Blood samples were drawn by nurses at each nurse visit. Participants were asked not to eat or drink for 5 hours and then their fasting blood samples were collected. Those who had clotting or bleeding disorders or taken anticoagulant drugs were ineligible to provide blood samples. Serum CRP was analyzed using the N Latex CRP mono immunoassay on the Behring Nephelometer II analyzer by The Department of Clinical Biochemistry at the Royal Victoria Infirmary (NewCastle-upon-Tyne, UK).

### Covariates

Age, sex, educational level, cigarette smoking, alcohol drinking, body mass index (BMI), cardiovascular diseases (CVD including angina, congestive heart failure and stroke), chronic lung diseases, cancer and dementia were considered as confounders. Educational level was categorized as high, intermediate and low level [35]. Cigarette smoking was classed as current smoking and non-smoking [36]. Frequency of alcohol consumption was measured in the last 12 months and grouped as daily, weekly/monthly, rarely/never [37]. The chronic conditions including CVD, chronic lung diseases, cancer and dementia were dichotomized as “yes” versus “no” based on self-reported physician diagnoses.

### Statistical analysis

**Fig. 1:**
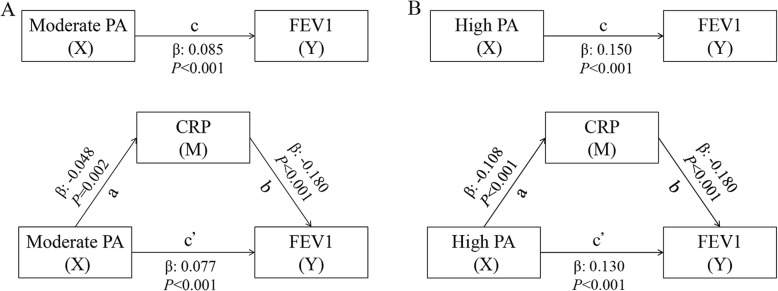
Results of mediation analysis for PA, CRP and FEV1. A for moderate PA; B for high PA. CRP: C-reactive protein; FEV1: forced expiratory volume in 1 second; PA: physical activity; β: regression coefficient. X: independent variable (cause); Y: dependent variable (outcome); M: mediator. Path a: X is related to M; Path b: M is related to Y after adjusting for X; Path c: X is related to Y; Path c^**’**^: X is related to Y when M is included in the models as a covariate. Models were adjusted for age, sex, education level, cigarette smoking, alcohol drinking, body mass index, cardiovascular diseases (angina, congestive heart failure and stroke), chronic lung diseases, cancer and dementia.

First, characteristics of participants were described using means ± standard deviations or percentages. The value of CRP was log-transformed in our analysis due to its markedly skewed distribution. Comparison of three PA groups were conducted by using ANOVA for normally distributed continuous data, Kruskal-Wallis H tests for ordinal data, and chi-square tests for categorical data. Second, Baron and Kenny’s causal steps method [38] was used to explore the possible associations among PA, CRP and lung function. In this method, X (PA) indicates independent variable, Y (lung function) indicates dependent variable, M (CRP) indicates mediator. Partial mediation is considered to have occurred if (1) X is related to Y (Fig. 1. Path c), (2) X is related to M (Fig. 1. Path a), (3) M is related to Y after adjusting for X as a covariate (Fig. 1. Path b), (4) the association between X and Y is significantly decreased when M is included in the models as a covariate (Fig. 1. Path c^**’**^). Third, the mediating effects were assessed by using linear regression models based on the Karlson/Holm/Bree (KHB) method [39]. This method estimated the direct, indirect (mediated), and total effects of PA on lung function and calculated the percentage of the main association explained by the mediator. All of the multiple regression models were developed adjusting for the potential confounders: age, sex, education level, cigarette smoking, alcohol drinking, BMI, CVD, chronic lung diseases, cancer and dementia.

To ensure the stability of the results, we conducted the sensitivity analysis by excluding participants who had chronic lung diseases and CVD.

Statistical analyses were performed using Stata version 15.0 (Stata Corp LP, College Station, TX). All *P*-values were two sided with a statistically significant level at 0.05.

## Results

Characteristics of the sample grouped by PA levels were listed in Table 1. A total of 6875 individuals were included in this study. Among them, 28.4% were defined as low PA, 49.8% were classified into moderate PA, and 21.8% into high PA. Univariate analyses showed that both CRP levels and lung function were significantly different across the PA groups (*P* < 0.001). Participants with higher PA levels had lower levels of CRP and better lung function.

**Table 1:** Characteristics of the participants by leisure-time physical activity levels (*n* = 6875)

Characteristics	Low PA	Moderate PA	High PA	*P*-value
	(*n* = 1952)	(*n* = 3422)	(*n* = 1499)	
Age, years, (Mean ± SD), (*n* = 6875)	69.18 ± 9.78	66.15 ± 8.60	63.76 ± 7.84	<0.001
Sex, males, (%), (*n* = 6874)	39.5	45.0	54.5	<0.001
Educational level, (%), (*n* = 6844)				
Low	35.3	20.2	12.9	<0.001
Intermediate	35.1	36.7	32.5	
High	29.6	42.1	54.6	
Smoking, (%), (*n* = 6875)				
Non-smoking	84.4	88.7	93.6	<0.001
Current smoking	15.6	11.3	6.4	
Alcohol intake, (%), (*n* = 6292)				
Rarely/Never	31.9	18.6	12.1	<0.001
Weekly/Monthly	55.2	67.0	71.0	
Daily	12.9	14.5	16.9	
BMI, kg/m^2^, (Mean ± SD), (*n* = 6655)	29.51 ± 5.79	27.99 ± 4.86	27.04 ± 4.33	<0.001
CRP, mg/L, (Mean ± SD)^a^, (*n* = 5410)	0.34 ± 0.47	0.20 ± 0.45	0.07 ± 0.44	<0.001
CVD, (%), (*n* = 6875)	15.6	6.6	4.2	<0.001
Lung diseases, (%), (*n* = 6875)	9.0	3.2	2.2	<0.001
Cancer, (%), (*n* = 6875)	5.7	5.0	4.1	0.084
Dementia, (%), (*n* = 6875)	1.4	0.4	0.3	<0.001
FEV1, L, (Mean ± SD), (*n* = 6875)	2.09 ± 0.74	2.46 ± 0.71	2.74 ± 0.74	<0.001
FVC, L, (Mean ± SD), (*n* = 6875)	2.93 ± 0.95	3.41 ± 0.93	3.75 ± 0.97	<0.001

*BMI* Body mass index, *CRP* C-reactive protein, *CVD* Cardiovascular disease (angina, congestive heart failure and stroke), *FEV1* Forced expiratory volume in 1 second, *FVC*: forced vital capacity, *PA* Physical activity, *SD* Standard deviation

^a^CRP was log-transformed due to its markedly skewed distribution

**Fig. 2:**
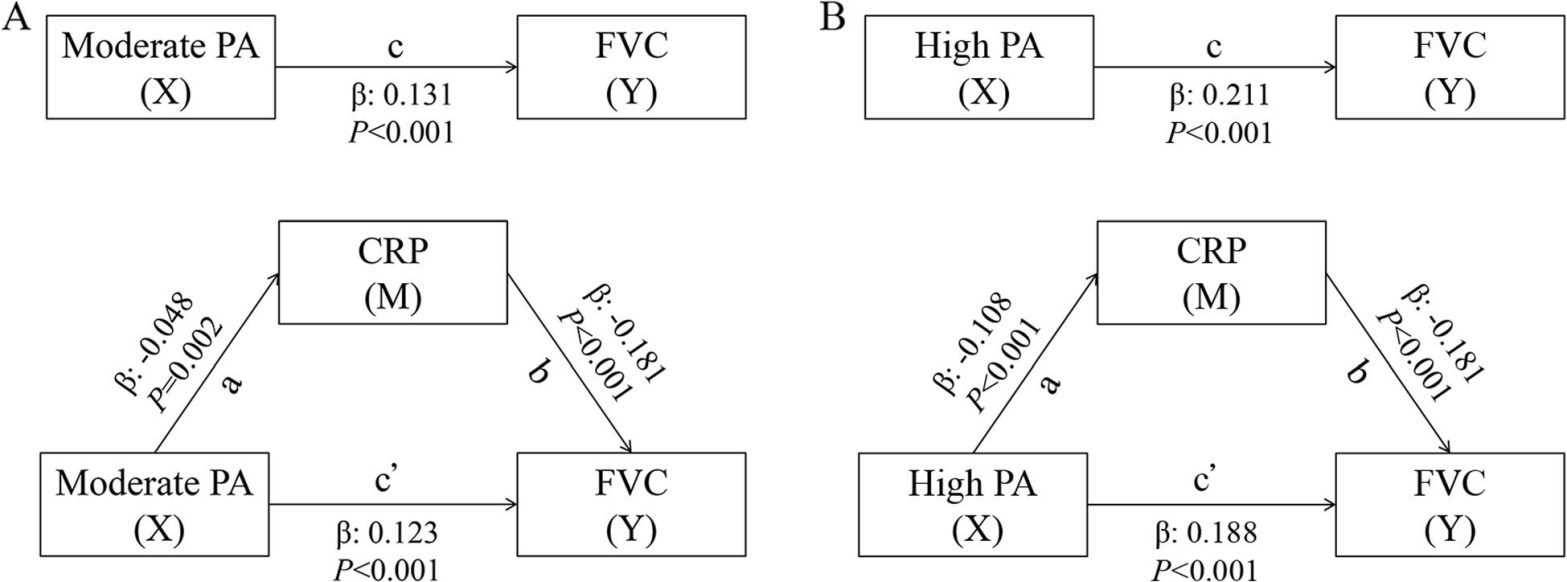
Results of mediation analysis for PA, CRP and FVC. A for moderate PA; B for high PA. CRP: C-reactive protein; FVC: forced vital capacity; PA: physical activity; β**:** regression coefficient. X: independent variable (cause); Y: dependent variable (outcome); M: mediator. Path a: X is related to M; Path b: M is related to Y after adjusting for X; Path c: X is related to Y; Path c^**’**^: X is related to Y when M is included in the models as a covariate. Models were adjusted for age, sex, education level, cigarette smoking, alcohol drinking, body mass index, cardiovascular diseases (angina, congestive heart failure and stroke), chronic lung diseases, cancer and dementia.

Results from the multiple linear regression analyses based on Baron and Kenny’s causal steps method were showed in Fig. 1 and Fig. 2. For Path a, compared with low PA, both moderate (β = − 0.048, *P* = 0.002) and high PA (β = − 0.108, *P* < 0.001) were associated with lower CRP levels. For Path b, CRP negatively correlated with FEV1 (β = − 0.180, *P* < 0.001) and FVC (β = − 0.181, *P* < 0.001) after adjustment for all covariates as well as PA. For Path c, PA showed positive effect on FEV1 (β = 0.085, *P* < 0.001 for moderate PA; β = 0.150, *P* < 0.001 for high PA) and FVC (β = 0.131, *P* < 0.001 for moderate PA; β = 0.211, *P* < 0.001 for high PA). After introducing the CRP into the models, regression coefficients of PA with FEV1 (β = 0.077, *P* < 0.001 for moderated PA; β = 0.130, *P* < 0.001 for high PA) and FVC (β = 0.123, *P* < 0.001 for moderated PA; β = 0.188, *P* < 0.001 for high PA) decreased (Path c^**’**^), indicating the potential mediating effects of CRP on the association between PA and lung function.

Table 2 displayed all the adjusted results of the mediation analysis based on the KHB method. The total effect, direct effect and indirect effect of high PA on lung function were statistically significant. After adjustment for the potential confounders, the indirect effect of high PA on FEV1 via CRP was 0.019 (*P* < 0.001) with 12.99% of the total effect being mediated; the indirect effect of high PA on FVC via CRP was 0.020 (*P* < 0.001) with 9.42% of the total effect being mediated.

**Table 2:** Direct and indirect effects of leisure-time physical activity with lung function

Lung function	Association	
	β (95%CI)	Mediation (%)^a^
FEV1		
PA-Moderate		
Total	0.085 (0.049, 0.121)	
Direct	0.077 (0.041, 0.113)	
Indirect via CRP	0.009 (−0.001, 0.017)	10.05
PA-High		
Total	0.149 (0.106, 0.193)	
Direct	0.130 (0.086, 0.174)	
Indirect via CRP	0.019 (0.010, 0.029)	**12.99**^**b**^
FVC		
PA-Moderate		
Total	0.131 (0.086, 0.176)	
Direct	0.123(0.078, 0.168)	
Indirect via CRP	0.009 (−0.001, 0.017)	6.58
PA-High		
Total	0.207 (0.153, 0.262)	
Direct	0.188 (0.133,0.243)	
Indirect via CRP	0.020 (0.010, 0.029)	**9.42**^**b**^

*CRP* C-reactive protein, *CI* Confidence interval, *FEV1* Forced expiratory volume in one second, *FVC* Forced vital capacity, *PA* Physical activity; β: regression coefficient

Models were adjusted for age, sex, education level, cigarette smoking, alcohol drinking, body mass index, cardiovascular diseases (angina, congestive heart failure and stroke), chronic lung diseases, cancer and dementia

Total effect is the effect of PA on lung function without CRP; direct effect is the effect of PA on lung function when controlling for CRP; indirect effect is the effect of PA on lung function via CRP

^a^Mediation (%) is calculated by indirect effect/total effect ×100

^b^*P*<0.001

Sensitivity analysis excluding participants who reported chronic lung diseases and CVD yielded results similar to those of the primary analyses (Additional file 1).

## Discussion

This large cross-sectional study in community-dwelling people documented that moderate and high PA were positively associated with lung function and were negatively associated with CRP levels. Higher CRP was independently related to poorer lung function. According to Baron and Kenny’s causal steps method and KHB method, CRP, as a sensitive marker of systemic inflammation, mediated the association between high PA and lung function.

Our findings on the positive effect of PA on lung function are consistent with previous studies. A prospective study in European adults aged 20–44 years founded that vigorous leisure-time PA was associated with higher FEV1 and FVC [13]. A longitudinal study involving 6790 participants reported that moderate to high levels of regular PA were associated with reduced lung function decline [12]. The similar results were also reported in a 10-year follow-up study in 8047 Norwegian men and women [14]. One of commonly accepted mechanisms underlying the association between PA and lung function is that PA can reduce inflammation damage in airways and thus prevent the decline of lung function [12, 16]. The beneficial effects of PA on lung function may be partially mediated by inflammation.

As with our finding, a cohort study in 5030 adults showed that PA played an important role in the attenuation of CRP levels [20]. Another 10-year follow-up study involving 4289 participants from the Whitehall II cohort study found regular PA was associated with lower inflammatory markers such as CRP and interleukin (IL)-6 [23]. Furthermore, a review which provided evidences from both cross-sectional and longitudinal investigations have demonstrated PA lowers CRP levels in a dose-response manner [25]. It is increasingly recognized that regular PA has long-term systemic anti-inflammatory effects [40]. Contracting skeletal muscle produces transiently a large dose of cytokines, such as IL-4, IL-10, and transforming growth factor-β (TGF-β) which not only have anti-inflammatory effects but also restrain the production of proinflammatory cytokines IL-6 and tumor necrosis factor-α (TNF-α) etc. [17]. Thus, it is possible that regular (long-term) exercise training could lead to lower basal levels of circulating inflammatory markers such as high-sensitivity CRP and reduced inflammatory response during acute exercise [20, 23].

Meanwhile, a longitudinal population-based study found the inverted effect of CRP on lung function in men [27]. Another prospective community-based study of a cohort showed that higher levels of CRP were associated with reduced lung function in young adults, suggesting that low-grade systemic inflammation may lead to impaired lung function [28]. In line with them, our results displayed the inverse association between serum CRP and lung function in middle-aged and older adults. There are increasing evidences that inflammatory mediators such as CRP and IL-6 in serum can activate pulmonary inflammatory cells in pulmonary circulation, leading to changes in pulmonary capillary endothelial function and the increases of pulmonary vascular filtration [19, 41]. Pulmonary microfiltration and systemic inflammation may result in damage in airways and accelerate the decline of lung function.

To our knowledge, this study is the first to characterize the relationship between PA, inflammation and lung function, by focusing on the role of inflammation in mediating PA and lung function. As expected, we found the inverse association between PA and CRP, as well as the negative effect of CRP on lung function. In addition, when serum CRP was introduced in the linear models as a covariate, PA decreased their effects on lung function, indicating the partial mediation of CRP. Further mediating effect analyses based on KHB methods showed that the indirect effect of high PA on lung function via CRP was statistically significant. Based on this epidemiological data, we found the role of inflammation as a mediator of the association between PA and lung function at the population-level. The increasing PA levels result in lower levels of circulating inflammatory markers, thereby protecting the small airways from inflammatory damage and preventing a decline in lung function.

A notable strength of this study was the relatively large number of participants in this large population-based study, which gave our statistical analyses sufficient power. Besides, in the sensitivity analysis, we excluded participants who had lung diseases and CVD to avoid reverse causation, because people with chronic diseases are likely to have high CRP levels in their serum and low PA. Some limitations should be considered. Firstly, our study is a cross-sectional design which lacks the direction of causality and gives little information to draw conclusions about long-term effects among PA, CRP and lung function. Secondly, PA were self-reported in this study, which may introduce measurement errors especially the reporting bias [42]. In addition, the current questionnaire for leisure time PA does not substantially address the issues of PA volume and might introduce error regarding PA levels. For example, some individuals may perform a large volume of low intensity activity and be classified ‘less’ active than someone who performs 2 very short bouts of moderate level activity. Thirdly, PA may be part of a healthy lifestyle, including intake of fresh fruit and vegetables, non-smoking and non-drinking, which may partly influence lung function. However, after adjusting for BMI, smoking and drinking as confounders, PA still had a positive effect on FEV1 and FVC. Finally, since CRP is a marker of systemic inflammation, future studies should explore whether these results are also suitable for the other inflammatory markers, such as IL-6. Yet, these results may hopefully stimulate interest in better understanding the relationships between PA and lung function.

## Conclusions

In conclusion, our results indicated that participants with increased PA levels had lower levels of serum CRP, in turn, kept better lung function. CRP as a sensitive marker of systemic inflammation mediated the association between PA and lung function. In the view of public health, these findings are potentially significant in the planning of preventive intervention strategies and physical activity-based programs, which aimed at reducing the systemic inflammation and promoting health.

## Supplementary information

**Additional file 1.** Sensitivity Analysis: Direct and indirect effects of leisure-time physical activity with lung function.

## Data Availability

The ELSA data are available from the UK Data Service (accession GN 33368) - https://www.ukdataservice.ac.uk/. This research uses data from the English Longitudinal Study of Ageing (ELSA). ELSA was developed by a team of researchers based at the NatCen Social Research, University College London and the Institute for Fiscal Studies. The data were collected by NatCen Social Research.

## Abbreviations

BMI: Body mass index
CRP: C-reactive protein
CVD: Cardiovascular diseases
ELSA: English Longitudinal Study of Ageing
FEV1: Forced expiratory volume in 1 second
FVC: Forced vital capacity
IL-10: Interleukin (IL)-10
IL-4: Interleukin (IL)-4
IL-6: Interleukin (IL)-6
KHB: Karlson/Holm/Bree
PA: Physical activity
TGF-β: Transforming growth factor-β
TNF-α: Tumor necrosis factor-α
UK: United Kingdom
