# Supplementary material for: C-reactive protein mediates the association between leisure-time physical activity and lung function in middle-aged and older adults
Source: BMC Public Health. 2020 Jan 6;20:6. doi: 10.1186/s12889-019-8028-y (PMC6945486; doi:10.1186/s12889-019-8028-y)
Supplement: Supplementary file 1 — Additional file 1. Sensitivity Analysis: Direct and indirect effects of leisure-time physical activity with lung function. [file 12889_2019_8028_MOESM1_ESM.docx]

**Additional file 3. Sensitivity Analysis: Direct and indirect effects of leisure-time physical activity with lung function**

| **Lung function** |  | **Association** | | | | |
| --- | --- | --- | --- | --- | --- | --- |
|  |  | **Primary analysis (n =6875)^a^** |  |  | **Sensitivity analysis (n = 6021)^b^** |  |
|  |  | **β (95%CI)** | **Mediation (%)^c^** |  | **β (95%CI)** | **Mediation (%)^c^** |
| **FEV1** | **PA-Moderate** |  |  |  |  |  |
|  | Total | 0.085 (0.049 , 0.121) |  |  | 0.097 (0.059 , 0.135) |  |
|  | Direct | 0.077 (0.041 , 0.113) |  |  | 0.088 (0.050 , 0.126) |  |
|  | Indirect via CRP | 0.009 (-0.001 , 0.017) | 10.05 |  | 0.009 (-0.001 , 0.018) | 8.82 |
|  | **PA-High** |  |  |  |  |  |
|  | Total | 0.149 (0.106 , 0.193) |  |  | 0.156 (0.110 , 0.201) |  |
|  | Direct | 0.130 (0.086 , 0.174) |  |  | 0.137 (0.091 , 0.182) |  |
|  | Indirect via CRP | 0.019 (0.010 , 0.029) | **12.99**^d^ |  | 0.019 (0.009 , 0.029) | **12.31**^d^ |
| **FVC** | **PA-Moderate** |  |  |  |  |  |
|  | Total | 0.131 (0.086 , 0.176) |  |  | 0.147 (0.099 , 0.195) |  |
|  | Direct | 0.123(0.078 , 0.168) |  |  | 0.138 (0.090 , 0.186) |  |
|  | Indirect via CRP | 0.009 (-0.001 , 0.017) | 6.58 |  | 0.008 (-0.001 , 0.017) | 5.70 |
|  | **PA-High** |  |  |  |  |  |
|  | Total | 0.207 (0.153 , 0.262) |  |  | 0.218 (0.160 , 0.275) |  |
|  | Direct | 0.188 (0.133 ,0.243) |  |  | 0.199 (0.141 ,0.256) |  |
|  | Indirect via CRP | 0.020 (0.010 , 0.029) | **9.42**^d^ |  | 0.019 (0.009 , 0.029) | **8.65**^d^ |

CRP: C-reactive protein; CI: confidence interval; FEV1: forced expiratory volume in one second; FVC: forced vital capacity; PA: physical activity; β**:** regression coefficient

^a^ Models were adjusted for age, sex, education, smoking, alcohol intake, body mass index, cardiovascular diseases (angina, congestive heart failure and stroke), chronic lung diseases, cancer and dementia.

^b^ Models were adjusted for age, sex, education, smoking, alcohol intake, body mass index, cancer and dementia.

Total effect is the effect of PA on lung function without CRP; direct effect is the effect of PA on lung function when controlling for CRP; indirect effect is the effect of PA on lung function via CRP.

^c^ Mediation (%) is calculated by indirect effect/total effect ×100.

^d^ P<0.001
